# The Ribonuclease A Superfamily in Humans: Canonical RNases as the Buttress of Innate Immunity

**DOI:** 10.3390/ijms17081278

**Published:** 2016-08-05

**Authors:** Patrick Koczera, Lukas Martin, Gernot Marx, Tobias Schuerholz

**Affiliations:** 1 Department of Intensive Care and Intermediate Care, University Hospital Rheinisch-Westfälische Technische Hochschule (RWTH) Aachen, Aachen 52074, Germany; pkoczera@ukaachen.de (P.K.); lmartin@ukaachen.de (L.M.); gmarx@ukaachen.de (G.M.); 2 Department for Experimental Molecular Imaging, University Hospital RWTH Aachen and Helmholtz Institute for Biomedical Engineering, RWTH Aachen University, Aachen 52074, Germany

**Keywords:** human RNases, canonical RNases, secreted RNases, host defence protein, antimicrobial activity

## Abstract

In humans, the ribonuclease A (RNase A) superfamily contains eight different members that have RNase activities, and all of these members are encoded on chromosome 14. The proteins are secreted by a large variety of different tissues and cells; however, a comprehensive understanding of these proteins’ physiological roles is lacking. Different biological effects can be attributed to each protein, including antiviral, antibacterial and antifungal activities as well as cytotoxic effects against host cells and parasites. Different immunomodulatory effects have also been demonstrated. This review summarizes the available data on the human RNase A superfamily and illustrates the significant role of the eight canonical RNases in inflammation and the host defence system against infections.

## 1. Introduction

The protein ribonuclease A (RNase A) was initially extracted from the bovine pancreas and is one of the best-characterized mammalian proteins in the literature. Over time, several other proteins with significant sequence homology were identified in mammals and other vertebrates, allowing assembly of the vertebrate-specific RNase A superfamily [1,2]. The members of this superfamily can be distinguished from other exo- and endoribonucleases, which show different distributions and properties [3]. In humans, eight secreted RNases have been described and are generally referred to as the canonical RNases. The following paragraph will highlight selected common features of these RNases with regard to their sequences, conformations, phylogenesis, biochemical characterization and regulation.

The proteins show a tertiary structure that is stabilized by eight disulphide bridges, with the exception of RNase 5, which has six cysteine residues. Two histidine residues and one lysine residue determine the catalytic activity of these RNases; the lysine residue lies within the common invariant sequence motif CKxxNTF. Each RNase initially contains an N-terminal signal sequence that directs protein biosynthesis within the endoplasmic reticulum, with its final form being secretory. Moreover, the N-terminal portion of the mature extracellular RNase appears to be required for antimicrobial activity [4]. This feature was demonstrated by generating N-terminus-derived peptides that showed similar antimicrobial activity. The ribonucleolytic activity, in contrast, appears to not be crucial for the activity against microbes [5].

The antibacterial activity of this protein family has been best characterized based on RNase 3 and is associated with disruption of the bacterial membrane. However, the mechanism of activity against viruses, fungi and parasites has not yet been resolved. Regarding antiviral activity, targeting of the virion is hypothesized. In addition to targeting the virion, the intracellular activity of these proteins in the cytosol might degrade viral RNA to inhibit viral replication or may induce host cell apoptosis [6,7]. As part of the Human Genome Project, corresponding genes were found to be located on chromosome 14, within cluster 14q11.2. Five additional proteins with relevant sequence similarity did not show ribonuclease activity or the characteristic N-terminal signal sequence [4,8]. The phylogenetic origin of the RNase A superfamily was extensively evaluated and further assessed with respect to the genome sequences of additional species. As a result, RNases 2 and 3 can be grouped together, as can RNases 7 and 8. Together with RNase 6, these RNases seem closely related. Meanwhile, RNases 1, 4 and 5 can be grouped, with a closer relationship between RNases 1 and 4 [9,10,11].

There is further information on the ancestral origin and role of this superfamily. While results regarding the function of RNases in zebrafish (*Danio rerio*) suggest an ancestral origin consisting of angiogenesis-related ribonucleases, a host defence-associated role was proposed in studies on birds and mammals [12,13,14,15]. This hypothesis was based on the proteins’ structure, biochemical properties and bactericidal functions.

In comparison to other immune-associated proteins/genes, the RNase A superfamily also exhibits high rates of duplication and amino acid substitution. Additionally, these RNases have high isoelectric points and positive net charges (with theoretically calculated pIs ranging from 8.69–10.12) [8,16]. Both properties are associated with antibacterial activity and strong interactions with the respective substrates of the enzyme reaction, namely, negatively charged polynucleotides. For the canonical RNases, each varies in ribonuclease activity and nucleotide preference for substrate cleavage, without strict selectivity for cleavage and recognition sites [10].

Different patterns of regulation of RNases, e.g., on the level of transcription or secretion, have been recognized. Notably, the ribonuclease inhibitor (RI), which can be found in all mammalian cells, controls the activity of all RNases in different ways. The RI binds to ribonucleases with femtomolar affinity and inhibits or attenuates the biological effects of the RNases by generating an RNase:RI complex. The presence of cytosolic RI protects the host cells from the cytotoxic activity of RNases [17].

Although the biochemical properties of RNase 1 and the emergence of the RNase A gene superfamily have been evaluated extensively, the RNases’ physiological function needs further clarification. To date, different reports have suggested the relevance of the RNases to host defence, angiogenesis and digestion. The present review summarizes information regarding the significance of the canonical RNases for human host defence (Table 1) and aims to provide a synopsis of current data with regard to the antimicrobial activity of the RNases and their roles in host immune responses.

## 2. Ribonuclease (RNase) 1

**Figure 1:**
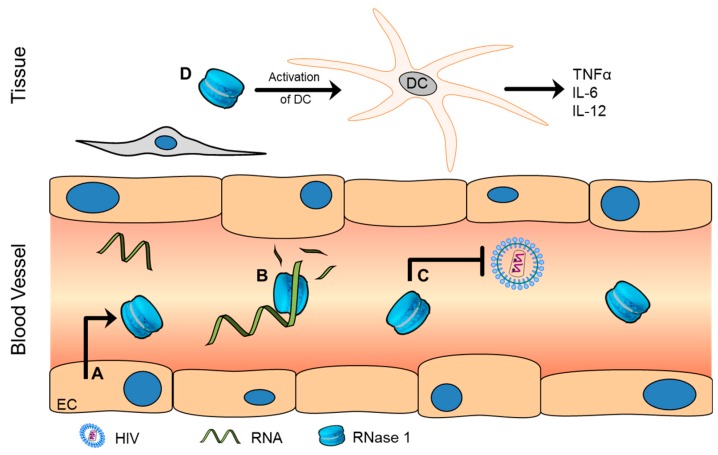
Ribonuclease (RNase) 1 is required for vascular homeostasis. (**A**) Endothelial cells (ECs) are the main source of large amounts of RNase 1; (**B**) Due to the ribonuclease activity of single- and double-stranded RNA as well as DNA-RNA hybrids, RNase 1 serves as a potent RNA scavenger for the normalization of serum viscosity and the clearance of perivascular polynucleotides; RNase 1 (**C**) shows antiviral activity against HIV and (**D**) is able to stimulate and induce maturation of dendritic cells (DCs). TNFα: Tumour necrosis factor alpha; IL: Interleukin; HIV: Human immunodeficiency virus; RNA: Ribonucleic acid.

RNase 1, also known as RNase A or pancreatic-type RNase, can be found in various organs, so its expression is not restricted to the exocrine pancreas [65]. This protein is known to undergo different post-translational modifications, and purified samples from the urine, seminal plasma, kidney and brain show different patterns of glycosylation [66,67,68,69]. The proposed functions of RNase 1 in immune defence are summarized in Figure 1. When taking into account the origin of these different modifications, the endothelial cells of the circulatory system appear to be one possible source, as they selectively produce and secrete RNase 1 in reasonable amounts, as demonstrated in vitro in human endothelial cells derived from veins, arteries and capillaries. Interestingly, human umbilical vein endothelial cells expressed and released the highest concentrations of RNase 1. Although endothelial cells constitutively secrete RNase 1, a fraction is stored in Weibel-Palade bodies, which are also known for storage and induced release of von Willebrand factor [70,71].

In ruminants, the RNase 1 secreted by the exocrine pancreas degrades dietary RNA to aid in nutrition, whereas in humans, digestion is not the main function [1,72]. The endothelial origin suggests association of this protein with vascular homeostasis [2]. RNase 1 has distinct ribonuclease activity, which allows degradation of single- and double-stranded polyRNA as well as DNA-RNA hybrids. Extracellular RNA is known as a dangerous molecule that can induce coagulation and endothelial permeability as well as modulation of the inflammatory response, partly through liberation of cytokines [18,73,74,75,76]. Therefore, RNase 1 is a potent polynucleotide scavenger that serves as an opposing force to vascular RNA in terms of coagulation, endothelial permeability and inflammation. Additionally, RNase 1 might be important for normalization of serum viscosity and clearance of perivascular pathogenic polynucleotides [70]. Although experimental data on humans are limited, the importance of extracellular RNA and RNase 1 has been illustrated very recently in different studies of animals [19]. Clinical proof of this concept was demonstrated by Cabrera-Fuentes et al. [77], who utilized upper-limb ischaemia for remote ischaemic preconditioning before cardiac surgery to protect the heart against ischaemia-reperfusion injury. By preconditioning, the blood levels of protective RNase 1 were increased, whereas vascular RNA and tumour necrosis factor α (TNFα) decreased. These findings indicate that enhancing the levels of RNase 1 by preconditioning before heart surgery may improve patient outcomes.

The relevance of RNase 1 to host defence is supported by reports of its antiviral activity. RNase 1 extracts from the urine for human chorionic gonadotropin preparations as well as recombinant RNase 1 showed antiviral activity against human immunodeficiency virus (HIV)-1, suggesting possible protection of the foetus during pregnancy [20,21,22]. Additionally, RNase 1 was demonstrated to induce activation and maturation of dendritic cells as well as subsequent production of different cytokines (e.g., TNFα, interleukin (IL)-6 and IL-12) [23]. Interestingly, for endothelial cells, studies have shown somewhat contradictory results. Following incubation with IL-1β or TNFα, endothelial cells were reported to have decreased secretion and cellular expression of RNase 1 due to an epigenetic mechanism [26]. These results indicate a disturbance of the vascular RNA/RNase system as a result of exposure to inflammatory stimuli.

## 3. RNase 2

RNase 2, commonly known as eosinophil-derived neurotoxin (EDN), can be found in the secondary granules of eosinophil granulocytes and is one of the four major secretory proteins released upon activation of eosinophils [27,28,29]. The discovery of this RNase is connected to the Gordon phenomenon, a non-physiological syndrome in rabbits that is characterized by cerebellar dysfunction upon intrathecal injection of EDN, as also shown in guinea pigs [78,79].

EDN shows antiviral activity against HIV and respiratory syncytial virus (RSV) in vitro [20,21,22,24,25]. Through cell culture experiments, it was shown that EDN reduces the infectivity of HIV and RSV. The ribonuclease activity of EDN appears to be crucial for the antiviral activity, as the RI eliminated the antiviral effect of activated eosinophils. Similarly, sequence-altered recombinant EDN lost its antiviral activity upon loss of ribonuclease activity [24]. However, the activation of EDN-producing eosinophil granulocytes is also associated with allergic inflammation, such as that related to asthma, in the respiratory tract. These two facets of eosinophil activation in the respiratory system could be considered as a side effect of host defence. The antiviral response against RSV by eosinophil granulocytes could be associated with immunization against allergen [80,81].

Eosinophils are not the only cells capable of EDN secretion; human monocyte-derived macrophages also produce this RNase upon stimulation with lipopolysaccharide (LPS) and TNFα [23]. Although EDN does not exhibit potent anthelmintic or antibacterial activity, eosinophils express different pattern recognition receptors, such as toll-like receptors (TLRs) and nucleotide-binding oligomerization domain (NOD)-like receptors, which determine interaction with bacteria and helminths [38,82,83]. Studies have reported the release of EDN upon incubation of eosinophils with pathogenic bacteria, such as *Clostridium difficile* or *Staphylococcus aureus*, but not with *Bifidobacteria*, *Hemophilus* or *Prevotella* species [30,31]. However, the specific mechanism of distinction between different bacteria needs further investigation. Yang et al. [32] described EDN as an alarmin because the ribonuclease was shown to facilitate antigen recognition by binding to TLR2 and stimulating a type 2 helper T (T_h_2)-polarized response. Further studies illustrated the effect of EDN on dendritic cells, with EDN acting as a chemoattractant for immature human dendritic cells. Furthermore, EDN was found to induce maturation and activation of cultured dendritic cells [23,33].

In summary, there is a strong need for further investigations into the role of EDN in host defence. Current thinking suggests that EDN causes elimination of cells by triggering an inflammatory response that activates killer cells or cell death pathways. Alternatively, EDN may be internalized by infected cells to degrade viral RNA in the cytoplasm or may be secreted by eosinophils or monocytes/macrophages to further modulate the immune response.

## 4. RNase 3

RNase 3, or eosinophil cationic protein (ECP), is another ribonuclease found in the secondary granules of eosinophils and is released upon cell activation/stimulation. Similar to EDN, ECP displays antiviral and neurotoxic activities, and anthelmintic, bactericidal and cytotoxic effects have also been described [8]. For example, incubation of ECP with target cell cultures reduced the infectivity of RSV group B. As with EDN, the antiviral activity of ECP is dependent upon its enzymatic, ribonucleolytic function. However, ECP’s bactericidal activity remains unaltered in enzymatically inactive RNase. With regard to the complex of the RI and ECP, the issue of pathogen toxicity is less clear. Data illustrating this topic are only available for parasites, as incubation of ECP with the RI suppresses the antiparasitic nature of the RNase [84,85]. Similar antiviral activity was described for EDN, and the antiviral activities of both ECP and EDN are dependent on their ribonuclease activity [34]. However, enzymatic function is not necessary for the antibacterial activity of ECP. Gram-positive and Gram-negative bacteria as well as certain mycobacterial strains (*Staphylococcus aureus*, *Escherichia coli* and *Mycobacterium vaccae*) have been shown to be susceptible to the antibacterial activity of ECP in vitro [35,36]. The mechanism of the antibacterial activity was demonstrated in recent studies, and a stepwise process was suggested. For Gram-negative bacteria, ECP appears to display an amyloid-like aggregation. ECP binds to the bacterial surface, which causes conformational alterations in the protein. This rearrangement of the protein enables bacterial agglutination by binding of the protein to other rearranged ECP molecules attached to bacterial surfaces. Finally, these ECP aggregates disrupt the LPS bilayer, which may result in membrane disruption and bacterial [36,86]. In this context, it is worth mentioning that the Alzheimer’s disease-associated amyloid β (Aβ) peptide has been discussed as a host defence strategy against fungal challenge, as amyloids possess antimicrobial properties [8,44,86,87,88,89,90,91]. These observations, although not completely understood, stress the role of ECP in bacterial clearance. As a result, further inflammatory pathways are initialized directly via the protein’s immunomodulatory effects, such as mast cell degranulation, and indirectly by the aforementioned antibacterial activity [37].

Eosinophils are associated with host defence strategies against helminthic parasites, and for granular proteins, ECP is the most potent anthelmintic member. ECP’s high toxic activity has been demonstrated in vitro against *Schistosoma mansoni* [38]. In vivo, epidemiological studies have illustrated the importance of ECP. The ECP gene shows sequence polymorphism, with the respective proteins differing in toxic activity against *Schistosoma mansoni.* Ugandan populations, which live in regions where the parasites are endemic, were tested for their distribution of ECP alleles. In this study, homozygous carriers of the more cytotoxic allele of ECP (434GG) showed a lower prevalence of *Schistosoma mansoni* infection. This finding indicates that the more cytotoxic variant of ECP improves elimination of *Schistosoma mansoni* infection. Additionally, another sequence polymorphism-related allele, 371G, was associated with a higher susceptibility to cerebral malaria, the most severe manifestation of infection with the parasite *Plasmodium falciparum* [39,40]. In addition, ECP showed toxic activity in other diseases caused by parasites, and specifically *Brugia pahangi* and *Trichinella spiralis* [41,42]. However, the specific mechanism needs further investigation.

**Figure 2:**
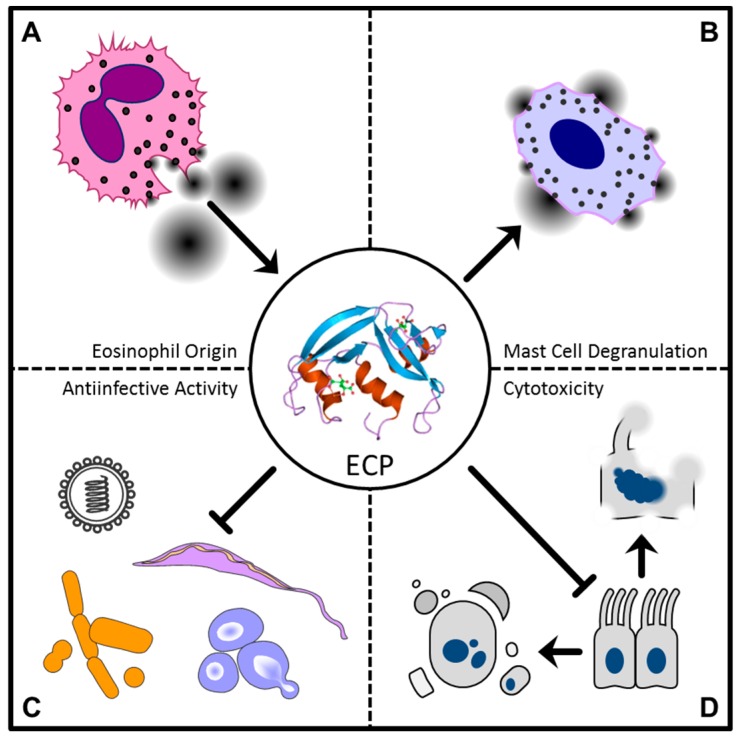
Effects of the eosinophil cationic protein (ECP). (**A**) ECP is stored in the secondary granules of eosinophils and can be released upon eosinophil simulation; (**B**) ECP can induce degranulation of mast cells; (**C**) ECP has broad antimicrobial activity, including inhibition of viruses, Gram-positive and Gram-negative bacteria and fungal and helminthic pathogens; (**D**) dose-dependent cytotoxic effects have been described for ECP, including necrosis and apoptosis.

Cytotoxic effects of ECP have been demonstrated against different host mammalian cell lines, including epithelial cells [43,44]. The process of the antimicrobial activity was reviewed previously [86,87], but further research needs to be performed to determine the significance of (host) cytotoxicity as an adverse effect of bactericidal, antiviral and anthelmintic activities or as a driver for tissue remodelling. In this regard, ECP has been shown to induce apoptosis in bronchial epithelial cells; however, the downstream mechanism of apoptosis needs further investigation. Intracellular as well as extracellular pathways were suggested for the apoptotic effect of ECP (heparan sulphate proteoglycan-mediated internalization for the former and cell surface aggregation for the latter), in addition to TNFα production and activation of the caspase-8 and caspase-3 pathways [92,93,94]. A summary of the activities of ECP is illustrated in Figure 2.

## 5. RNase 4

The significance of RNase 4 for physiological function in general and for host defence in particular is widely unknown because very few studies have been performed to investigate its function. RNase 4 mRNA was detected in several human somatic tissues, such as the skeletal muscle, pancreas, lung, kidney, placenta, liver and blood (i.e., in monocytes) [45,46,47]. The last two sources suggest a role in host defence, as these tissues play a significant role in this regard. No direct evidence for antimicrobial activity of RNase 4 in humans has been described to date, but two observations are worth noting. First, RNase 4 extracted from bovine milk reduced the viability of *Candida albicans* in vitro, and the antimicrobial effects of lactoferrin and lactoferricin against *E. coli* were enhanced by co-incubation with a mixture of RNases 4 and 5. Second, in vitro studies showed that the mRNA of RNase 4 was found in proliferating and differentiated human keratinocytes [48,95,96]. In these studies, RNase 4 expression was associated with the expression of RNase 5, another member of the RNase family with proven antimicrobial activity.

## 6. RNase 5

RNase 5, or angiogenin, was named after its angiogenic potency, which induces extensive blood vessel growth [97]. This angiogenic property requires ribonuclease activity, but in comparison to the enzymatic activity of RNase 1, RNase 5 has much lower activity levels (1 × 10^−5^–1 × 10^−6^) [8,98,99,100]. Both its enzymatic activity and its angiogenic effect are modulated by the RI. RNase 5 can be detected in different tissues and organs and is associated with a number of (patho) physiological processes, including neoplasia, reproduction and regeneration of damaged tissue [101,102,103]. The aforementioned processes include activation of both the immune system and angiogenesis, each happening at different stages, which hampers distinction between those processes. For RNase 5, the patterns of expression vary in comparison to those of other inducers, such as vascular endothelial growth factor. These observations hint at a broader physiological role for RNase 5 in addition to a significant role in inflammatory processes and host defence. The broad biological relevance of this protein was recently reviewed, with a focus on its function in host defence [100]. It was reported that serum levels of RNase 5 increase during acute-phase responses [49,50]. Similar to RNase 4, RNase 5 is secreted by proliferating and activated keratinocytes; in fact, in the cervical-vaginal lavage of women with a sexually transmitted disease, RNase 5 levels were elevated [48,51]. The antimicrobial and antiviral activities of RNase 5 were also demonstrated in vitro. RNase 5 inhibited the reproduction of HIV-1 and reduced the number of colony counts of *Streptococcus pneumoniae* and *Candida albicans* [22,52]. Although the latter observation has been questioned, research in other species supports the antimicrobial property of RNase 5 [53]. In bovine milk, RNase 5 showed antifungal activity against *Candida albicans*, and particularly the hyphal form. The antimicrobial effects of lactoferrin and lactoferricin against *E. coli* were enhanced by co-incubation with a mixture of RNases 4 and 5 [95,96]. Furthermore, the antifungal activity against *Candida albicans* depends on the ribonuclease activity because blocking the enzyme significantly reduced the capacity to kill *Candida* [48]. In addition, murine analogues of RNase 5 (Ang1 and Ang4) showed antimicrobial activity; Ang4 was secreted into the murine intestine by Paneth cells and goblet cells of the colon upon microbiological challenge with LPS or *Salmonella* species, for example [52,104,105].

The direct effects of RNase 5 on host defence cells have also been reported. Upon microbiological challenge, mast cells synthesize RNase 5 and store it in their granules. In particular, LPS, *E. coli* and peptidoglycans can induce the secretion of this protein. Incubation with RNase 5 stimulated leukocytes to synthesize pro-inflammatory cytokines such as IL-6 and TNFα. In contrast, degranulation of neutrophil granulocytes was inhibited by incubation with RNase 5, which in turn can inhibit hyper-inflammatory states during the immune response [54,55,56,57].

Together, these reports suggest that RNase 5 plays a role involving not only direct antimicrobial activity but also modulation of the immune response as part of the host defence system.

## 7. RNase 6

Little evidence for the significance of RNase 6 has been found to date. mRNA transcripts of this protein were found in different tissues, including those of the lung, heart, brain, placenta, liver, skeletal muscle, kidney and pancreas. Detection in neutrophil granulocytes and monocytes suggested a role in inflammation [106]. Very recently, disease-associated functions for RNase 6 were described. It was demonstrated that RNase 6 protein levels in the urinary tract were elevated upon infection in vivo. Monocytes/macrophages secreted RNase 6 upon *E. coli* challenge. The protein showed in vitro antimicrobial activity against different uropathogenic bacterial strains, including *E. coli*, *Staphylococcus saprophyticus* and *Enterococcus faecalis* [58]. The mechanism of this antimicrobial activity was evaluated recently. The protein is able to destabilize the membrane of Gram-negative bacteria and to agglutinate them [59]. As demonstrated for RNase 1, 2 and 5, also RNase 6 has impact on the HI virus. Incubation of RNase 6 with target cells *in vitro* inhibits HIV infection [107]. These findings highlight the role of RNase 6 for host defence.

## 8. RNase 7

RNase 7 was first detected and purified from human skin; in fact, it is the most abundant RNase found in the skin and is constitutively expressed. It has been proposed that this protein, together with additional components, such as the secreted antimicrobial proteins β-defensin and psoriasin, constitutes the host defence system of the cutaneous epithelia. In psoriatic lesions, the levels of these proteins are elevated. This finding suggests a possible cause for the low incidence of infections related to psoriatic lesions [48,108,109,110]. RNase 7 is also expressed by various epithelial tissues, such as the genitourinary tissues; respiratory tissues and, to a lesser extent, the cells of the gastrointestinal tract, and is associated with the host defence properties of these tissues in response to environmental and microbial challenges [60,61]. In skin, keratinocytes are the major source of RNase 7; the protein was shown to be secreted in combination with the RI. The proteolytic activity in the stratum corneum reveals the broad-spectrum antimicrobial activity of RNase 7 upon degradation of the RI [48]. For the urogenital tract, a distinct form of control was recently demonstrated, as shown by decreased RI expression upon infectious challenge causing pyelonephritis [48,60]. Although RNase 7 shows high constitutive expression in keratinocytes, the expression of RNase 7 in cultured primary keratinocytes can be further enhanced by incubation with TNFα, IL-1β, or interferon γ (IFNγ) or by microbial challenge with *Pseudomonas aeruginosa*, *E. coli*, *Staphylococcus aureus* and *epidermidis*, *Streptococcus pyogenes* or *Trichophyton rubrum*. It has also been demonstrated in vitro that cigarette smoke enhanced RNase 7 expression in the respiratory epithelium and that protozoan challenge with *Acanthamoeba castellanii* increased mRNA expression of RNase 7 in corneal epithelial cells [108,111,112]. In these studies, recognition and signal transduction were associated with the TLR2, epidermal growth factor receptor (EGFR), nuclear factor κ-light-chain enhancer of activated B cells (NFκB), signal transducer and activator of transcription (STAT) 3 and mitogen-activated protein kinase (MAPK) pathways [62,63,111,113]. In human umbilical vein endothelial cells, RNase 7 was induced upon incubation with inflammatory cytokines, including TNFα, or co-incubation with IL-1β and IFNγ, suggesting a role in host defence of the tissue-blood barrier [114]. Broad antimicrobial activity was demonstrated for RNase 7 against different bacterial strains and fungi, but antiviral activity has not yet been described. It was reported that the protein is active against both Gram-positive and Gram-negative bacteria of clinical interest (*E. coli*, *Enterococcus faecium*, *Pseudomonas aeruginosa* and *Staphylococcus aureus*) as well as against mycobacteria (*Mycobacterium vaccae*), which are emerging in the clinic. RNase 7 shows activity at micromolar concentrations, even against multidrug-resistant isolates of *Enterococcus faecium* (vancomycin-resistant *Enterococci*, or VRE). Additionally, fungi, such as *Candida albicans* or the dermatophyte *Epidermophyton floccosum*, are susceptible to RNase 7 [36,108,115,116]. The mechanism of the antimicrobial activity is not yet fully understood, but several studies have sought to address this question. The ribonuclease activity does not appear to be relevant to the antimicrobial activity; rather, it is proposed that RNase 7 has the ability to disrupt bacterial membranes. In contrast to the action of RNase 3, there is no agglutination of the bacteria. It has been reported that the cationic protein interacts electrostatically with synthetic lipid vesicles, which causes leakage of the spheres. It was also reported that the positively charged protein is capable of forming a complex with LPS as well as outer membrane protein I (OprI) from *Pseudomonas aeruginosa*. For this bacterium, the interaction causes permeation of the membrane and triggers cell death [64,117].

## 9. RNase 8

Although there is a strong sequence homology between RNase 7 and RNase 8, their distinct patterns of expression imply different roles. RNase 8 is expressed in the placenta and later on in the spleen, lung and testis [118]. However, its physiological function is still unknown. The antimicrobial activity of this protein has been demonstrated by its killing of different clinically relevant Gram-positive and Gram-negative bacteria, including different multidrug-resistant strains (methicillin-resistant *Staphylococcus aureus* and VRE), as well as *Candida albicans* [119]. The antimicrobial properties and the expression of RNase 8 in the placenta suggest its significance during host defence in pregnant women. This implies an additional defence system between the mother and her sterile foetus because different pathogens are known to pass through the placenta to the foetus [120].

## 10. Conclusions

**Figure 3:**
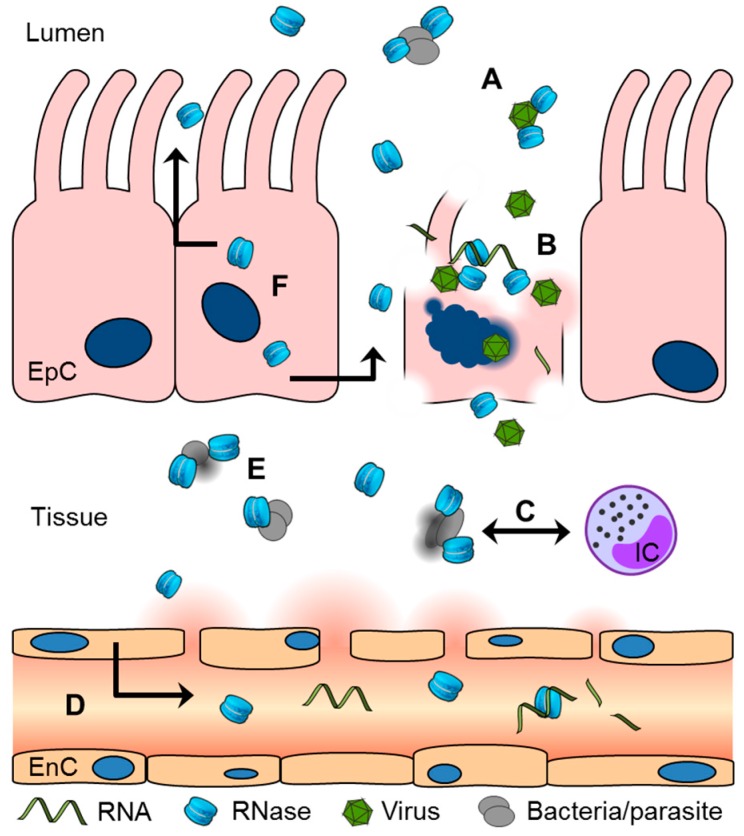
A schematic overview of the human canonical ribonucleases (RNases) in the host defence system. (**A**,**B**) RNases show antiviral activity and cytotoxic properties in mammalian cells and degrade RNA; (**C**) Cells of the immune system (immune cells, or ICs) secrete and are modulated by RNases; (**D**,**F**) Different mature cells secrete RNases, including endothelial (EnC) and epithelial (EpC) cells, for pericellular homeostasis; (**E**) Antimicrobial activity against bacteria, fungi and parasites has been demonstrated for RNases. RNA: Ribonucleic acid; HIV: Human immunodeficiency virus.

The scientific investigation of pancreatic RNase and the RNase A superfamily began with an evaluation of the biochemical properties of these proteins and was continued to obtain a better understanding of the biological significance of each family member in terms of homeostasis and pathophysiology. The canonical RNases are able to digest polynucleotides in addition to their antiviral, antibacterial, anthelmintic, antifungal and cytotoxic activities. They are secreted by different tissues and by cells of the immune system, so they also possess immunomodulatory properties (Figure 3) [8,121]. These findings demonstrate the significance of canonical RNases for human host defence in general and as a backbone of the innate immune system in particular. However, there is a strong need for further evaluation. In humans, RNases appear to strongly protect the body’s interfaces with the environment, which include the cutaneous, urogenital and respiratory epithelia. Information regarding the significant role of RNases in protection against infection and severe inflammatory diseases, such as sepsis, is very limited, but as recently shown by our group, these proteins appear to be important players in acute infections [122].

Another benefit to gaining a better understanding of secreted RNases is the pharmacological exploitation of this family of proteins. With the deluge of multidrug-resistant bacteria and a drought in the antibiotic pipeline, conventional antibiotics have lost some of their usefulness in the battle against infections. Together with antimicrobial peptides, for which resistance is less threatening, RNases could offer a new solution with concurrent use of conventional antibiotics [123]. Further research is needed to evaluate the usefulness of treating infections directly with RNases and of exploiting their antimicrobial activity for the creation of synthetic analogues for tailored applications.

## Figures and Tables

**Table 1:** Proposed functions of the canonical ribonucleases (RNases) in human host defence.

Ribonuclease	Proposed Impact on Host Defence	Reference(s)
RNase 1	Degradation of vascular polyRNA	[18,19]
	Anti-HIV-1 activity	[20,21,22]
	Induces maturation and activation of dendritic cells	[23]
RNase 2/EDN	Antiviral activity against HIV-1 and RSV-B	[20,21,22,24,25]
	Secretion by eosinophil granulocytes and monocyte-derived macrophages	[23,26,27,28,29,30,31]
	TLR2 binding and T_h_2 polarization	[32]
	Chemokine and cytokine induction for activation and maturation of dendritic cells	[23,33]
RNase 3/ECP	Antiviral activity against RSV-B	[34]
	Antibacterial activity against mycobacteria and Gram+ and Gram− bacteria	[35,36]
	Induces degranulation of mast cells	[37]
	Anthelmintic activity against *Schistosoma mansoni*, *Brugia pahangi* and *Trichinella spiralis*	[38,39,40,41,42,43]
	Cytotoxic activity against mammalian cells	[43,44]
RNase 4	Expression in host defence-associated tissues	[45,46,47,48]
	Coexpression with lactoferrin, lactoferricin and RNase 5 Enhances antimicrobial activity of lactoferrin and lactoferricin	
RNase 5/Angiogenin	Increased serum levels during acute-phase response	[49,50,51]
	Antiviral activity against HIV-1	[22]
	Activity against *Candida*	[48,52,53]
	Activity against *Streptococcus* (controversial data)	
	Synthesis and secretion by mast cells	[54]
	Proinflammatory stimulation of leukocytes	[55]
	Inhibition of degranulation of neutrophil granulocytes	[56,57]
RNase 6	Infection-induced secretion in urinary tract	[58,59]
	Antibacterial activity against Gram+ and Gram− bacteria	
RNase 7	Synthesis upon microbial, inflammatory and physicochemical challenge in epithelial tissues	[59,60,61,62]
	Antibacterial activity against mycobacteria and Gram+ and Gram− bacteria	[36,59,63]
RNase 8	Antibacterial and antifungal activity against Gram+ and Gram− bacteria and *Candida*	[64]

RNase: Ribonuclease; RNA: Ribonucleic acid; HIV-1: Human immunodeficiency virus 1; RSV-B: Respiratory syncytial virus B; EDN: Eosinophil-derived neurotoxin; TLR2: Toll-like receptor 2; T_h_2: Type 2 helper T-cell; ECP: Eosinophil cationic protein.

## Abbreviations

RNase A	Ribonuclease A
RI	Ribonuclease inhibitor
RNA	Ribonucleic acid
DNA	Deoxyribonucleic acid
HIV	Human immunodeficiency virus
RSV	Respiratory syncytial virus
TNFα	Tumour necrosis factor α
IL	Interleukin
EDN	Eosinophil-derived neurotoxin
LPS	Lipopolysaccharide
TLR	Toll-like receptor
NOD	Nucleotide-binding oligomerization domain
T_h_2	Type 2 helper T
ECP	Eosinophil cationic protein
*E. coli*	*Escherichia coli*
EGFR	Epidermal growth factor receptor
NFκB	Nuclear factor κ-light-chain enhancer of activated B cells
STAT	Signal transducer and activator of transcription
MAPK	Mitogen-activated protein kinase
IFNγ	Interferon γ
VRE	Vancomycin-resistant enterococci
OprI	Outer membrane protein I
